# Mapping Pain Memory Across Modalities: Distinct Spatial Representations of Recalled Visceral and Somatic Pain

**DOI:** 10.1002/ejp.70395

**Published:** 2026-09-21

**Authors:** Catrin Guddat, Genisius Hartanto, Robert J. Pawlik, Thomas Graven‐Nielsen, Sigrid Elsenbruch, Jana L. Aulenkamp

**Affiliations:** ^1^ Department of Neurology, Center for Translational Neuro‐and Behavioral Sciences (C‐TNBS) University Hospital Essen, University of Duisburg‐Essen Essen Germany; ^2^ Department of Medical Psychology and Medical Sociology, Center for Medical Psychology and Translational Neurosciences Ruhr University Bochum Bochum Germany; ^3^ Center for Neuroplasticity and Pain (CNAP), Department of Health Science and Technology Aalborg University Aalborg Denmark; ^4^ Department of Anesthesiology and Intensive Care Medicine University Hospital Essen, University of Duisburg‐Essen Essen Germany

## Abstract

**Background:**

Representation of pain areas offers a spatial dimension of pain perception, yet it has rarely been used in the context of visceral pain. This experimental study examined spatial patterns of acute visceral and somatic pain and their recall.

**Methods:**

Healthy volunteers (*N* = 49, 30 females) underwent calibrated rectal distension (visceral pain) and cutaneous heat stimulation on the abdomen (somatic pain), and drew modality‐specific pain areas on standardised body maps immediately after stimulation and one week later. Drawings were digitised and quantified, distinguishing local from referred pain, defined as pain area separated from local site. Pain intensity and unpleasantness were assessed with visual analogue scales, psychological traits and states via questionnaires.

**Results:**

Rectal distension induced larger and more diffuse areas than heat pain (*p* < 0.001). Pain areas were highly reproducible after one week (*p* < 0.001). Referred visceral pain was reported by 67% of participants, predominantly in the anterior abdomen, whereas somatic pain remained localised. Total pain areas were unrelated to pain intensity or unpleasantness. However, participants with referred visceral pain had higher trait anxiety (*p* = 0.011), while greater referred visceral pain extent was associated with higher pain intensity (*p* = 0.012). Bayesian analyses identified extraversion and behavioural inhibition as predictors of visceral pain area.

**Conclusion:**

Visceral and somatic pain showed distinct, reliably recalled spatial representations. Visceral pain was more extensive and diffuse. Its association with personality traits suggests that spatial pain representations reflect stable, trait‐like characteristics, highlighting the value of body maps as complementary tool for characterising interoceptive pain in experimental and clinical research.

**Significance Statement:**

Visceral pain evokes a broader and more diffuse spatial representation than somatic pain, which is reliably recalled and shaped by psychological traits. Body maps capture features such as referred pain that are not reflected by intensity ratings alone. Visceral pain was predicted by personality trait factors, while referred pain extent was linked to pain intensity. These findings support modality‐specific body maps as a mechanism‐informed tool for stratifying visceral interoceptive pain in research and clinical contexts.

## 1 Introduction

Visceral pain is a prevalent and burdensome symptom, characterised by unique perceptual qualities and strong association with autonomic responses that can cause additional symptoms such as nausea and profuse sweating (Drewes et al. 2020, 2002; Grundy et al. 2019). Compared with somatic pain, it is typically less localised, more diffuse and can evoke pronounced emotional and psychophysiological responses even at low intensity, including fear, negative expectations and hypervigilance, engaging brain networks relevant to affective pain processing and modulation (Aulenkamp et al. 2026; Chae et al. 2022; Dunckley, Wise, Aziz, et al. 2005; Koenen et al. 2017, 2021; Strigo et al. 2002; Van Oudenhove et al. 2020). These characteristics contribute to the clinical complexity of visceral pain, which in chronic conditions involving the gut‐brain axis, such as irritable bowel syndrome, arises from multifactorial mechanisms including sensitised peripheral and central mechanisms and autonomic nervous system contributions (Arendt‐Nielsen et al. 2018). Visceral pain is challenging to assess and manage, and commonly used tools often fail to capture its full perceptual characteristics (Grundy et al. 2019).

Body maps are valuable tools for documenting pain location and extent (Schott 2010; Shaballout et al. 2019). Unlike verbal descriptors or numerical rating scales, they quantify spatial characteristics of pain, allowing inferences about differences between pain modalities and between referred and local pain (Billot et al. 2025; Kwong et al. 2023). Recent studies indicate that pain body maps can help distinguish central from peripheral pain mechanisms, detect changes in pain patterns over time and across conditions, and explore psychological factors that modulate pain perception (Doménech‐García et al. 2016; Fishbain et al. 2003; Palsson et al. 2021). Referred pain is particularly relevant to visceral pain and occurs when nociceptive input from internal organs is perceived in somatic structures because visceral and somatic afferents converge in the spinal cord (Arendt‐Nielsen and Svensson 2001; Graven‐Nielsen 2006; Pedersen et al. 2004). Characterising both local and referred pain areas is therefore essential for understanding visceral pain mechanisms (Laursen et al. 1997).

Pain body maps remain underutilised in experimental visceral pain research, and direct within‐subject comparisons of visceral and somatic pain are scarce (Strigo et al. 2002). Most studies focused on musculoskeletal pain or other clinical populations (Doménech‐García et al. 2016; Lee et al. 2012; Pedersen et al. 2004); for example, muscle pain area strongly correlates with intensity and frequently produces referred pain (Graven‐Nielsen 2006).

The present analysis focussed on pain areas captured by body maps alongside complementary measures of acute visceral and somatic pain experience in healthy individuals, using controlled rectal distension and somatic heat stimuli applied to the same body region as well‐established experimental pain models. The primary objectives were to: (1) quantify and compare the size and spatial distribution of pain areas for visceral and somatic pain, including reproducibility based on recalled perception after one week; (2) examine associations between pain areas and pain ratings within and between pain modalities; (3) identify psychological traits predicting pain area; and (4) compare participants with and without referred pain, captured by body maps.

## 2 Methods

### 2.1 Study Setting and Participants

The comprehensive ‘*NOVIS*’ study was conducted at the University Hospital Essen, University of Duisburg‐Essen, Germany, within the Collaborative Research Centre (CRC) 289 ‘Treatment Expectation’ funded by the German Research Foundation. The study was approved by the Ethics Committee of the University Hospital Essen (19–8897‐BO), preregistered in the German Clinical Trials Register (DRKS00024410), and performed in accordance with the Declaration of Helsinki. All volunteers provided written informed consent and received monetary compensation. The conceptual background and full analysis plan are described in a published protocol (Aulenkamp et al. 2023), and primary outcomes of the full NOVIS trial have been reported previously (Aulenkamp et al. 2026).

The present manuscript provides results of the first analysis of spatial pain‐area data derived from body maps within the NOVIS project. Analyses were conducted in a clearly defined subsample of 49 healthy volunteers (30 women, 19 men; mean age 24.5 ± 3.1 years; mean body mass index 23.33 ± 2.64). Participants all received identical noxious stimulation intensities and timing across all experimental phases, described in detail below, ensuring that spatial pain characteristics were not influenced by experimental manipulations of stimulus strength but instead reflected inherent perceptual features of visceral and somatic pain. Participants were drawn from two groups that differed only in treatment suggestions: (1) a 50% probability of receiving a pro‐nociceptive drug versus saline, or (2) neutral information describing saline as inert. The absence of suggestion effects on all pain‐area and pain‐intensity measures was confirmed before pooling the groups.

Participants underwent an established screening process including structured interviews, medical examination, and questionnaires. Detailed information on inclusion and exclusion criteria are provided here (Aulenkamp et al. 2023, 2026). Briefly, the most important exclusion criteria included BMI < 18 or > 30 kg/m^2^, age < 18 or > 45 years, any ongoing medical condition or regular medication use, recent or current gastrointestinal or pain symptoms, any diagnosed pain‐ or GI‐related disorder, anal tissue abnormalities that could interfere with rectal distension, and psychiatric conditions or current symptoms of anxiety or depression, assessed via the German version of the Hospital Anxiety and Depression Scale (HADS) (Laux et al. 2013). Recent participation in any visceral or somatic pain study (past three months, based on self‐report) was also exclusionary to prevent prior pain experience from influencing the immediate or recalled experience of pain.

### 2.2 Study Design and Overview of Procedures

The study consisted of two laboratory sessions conducted one week apart and employed an established multiple threat paradigm involving a series of visceral pain induced by pressure‐controlled rectal distension and somatic pain induced by thermal cutaneous stimuli (Koenen et al. 2017, 2021). On study day 1, the multiple threat paradigm was implemented in three consecutive experimental phases: (i) a pre‐treatment baseline, (ii) a treatment phase in which intravenous saline infusion was combined with verbal suggestions, and (iii) a post‐treatment phase. On study day 2, participants were reinvited to the lab for a follow‐up pain session, yet without any treatment suggestion or physical assessments. All participants included in the present analysis underwent the identical multiple threat paradigm in all three experimental phases; hence, noxious stimulation protocols used the same number of stimuli, identical timing, and individually calibrated pain intensities applied consistently across all phases. Pain‐area data for visceral and somatic pain were collected at two time points: (1) At the end of study day 1, capturing immediate pain experienced during all three phases, and (2) at the beginning of study day 2, prior to re‐exposure to any pain stimuli, capturing delayed recall of pain experienced one week earlier. Visual analogue scale (VAS) ratings of perceived pain intensity and unpleasantness were collected at multiple time points following pain stimuli.

### 2.3 Pain Stimuli

Visceral pain was evoked using pressure‐controlled rectal distension generated by a barostat system (Distender Series II, G&J Electronics; 1300 mL single balloon), a widely established and clinically relevant model of experimental visceral pain (Keszthelyi et al. 2012). The balloon catheter, when inflated without any external resistance, was capable of expanding to a height of 10 cm and a diameter of 15 cm. However, when positioned in the rectum, this maximal expansion was not achieved, as the external counterpressure of the intestinal wall limited expansion and ultimately determined the pressure (in mmHg), which was fed back to the computer‐controlled barostat system. The balloon catheter was inserted such that the balloon extended from approximately 5 to 15 cm proximal to the anal sphincter.

For the somatic pain modality, thermal cutaneous heat pain was induced with a thermal stimulation device (PATHWAY model CHEPS; Medoc Ltd., Advanced Medical Systems, Ramat Yishai, Israel). The heated surface of the thermode measured 3 × 3 cm and was applied to the left lower abdomen. This site was chosen due to its anatomical proximity to the rectum, enabling both pain modalities to originate from a comparable body region. A key methodological requirement of the multiple threat paradigm employed herein constitutes the individualised calibration of stimulus intensities within predefined perceptual ranges and matched across pain modalities (Aulenkamp et al. 2023; Koenen et al. 2017). Before the first stimulation phase, a multistep procedure was used, including determination of pain thresholds, modality‐specific calibration and matching, and a brief habituation phase. Crucially, visceral and somatic stimuli were individually adjusted to elicit a target pain intensity of 50 mm on a 0–100 mm VAS (anchored with ‘not painful’ to ‘extremely painful’). Safety limits of 60 mmHg for distension and 49.5°C for heat stimulations were strictly observed. Final calibrated mean intensities herein were 32.0 ± 9.7 mmHg for distension and 45.9°C ± 1.37°C for heat stimuli, consistent with our findings in independent healthy samples (Koenen et al. 2021) and the full sample (Aulenkamp et al. 2026). Each noxious stimulation phase employed the identical series of cued visceral and somatic pain stimuli, comprising per phase a total of 12 phasic pain stimuli (i.e., 6 distensions, 6 thermal cutaneous stimuli). Stimuli were delivered in pseudorandomised order starting with a visceral or a somatic stimulus in 50% of participants, respectively. Visceral distension stimuli had a total duration of 20 s, including inflation, plateau and deflation phases, followed by a 55 s interval of no stimulation. Depending on the target pressure determined during pain calibration, the combined inflation and deflation rates ranged from 1 to 10 s, resulting in a plateau duration of 10 to 19 s. This was attributable to the physical properties of the pressure‐controlled barostat system. Due to greater flexibility in parameter settings of the somatic heat stimuli, these were adjusted such that their ramp‐up and ramp‐down phases, as well as the resulting plateau duration, were matched to the temporal characteristics of the visceral distension stimulus within each participant.

### 2.4 Pain Body Maps

Spatial pain characteristics were assessed using standardised paper‐and‐pencil body maps showing anterior and posterior torso views (see example in Aulenkamp et al. 2023). Participants shaded areas corresponding to their perceived pain immediately after study day 1 (recall of overall visceral and somatic pain experienced that day) and at the beginning of study day 2 (recall of pain experienced one week earlier). Separate maps were completed for visceral and somatic pain.

**FIGURE 1:**
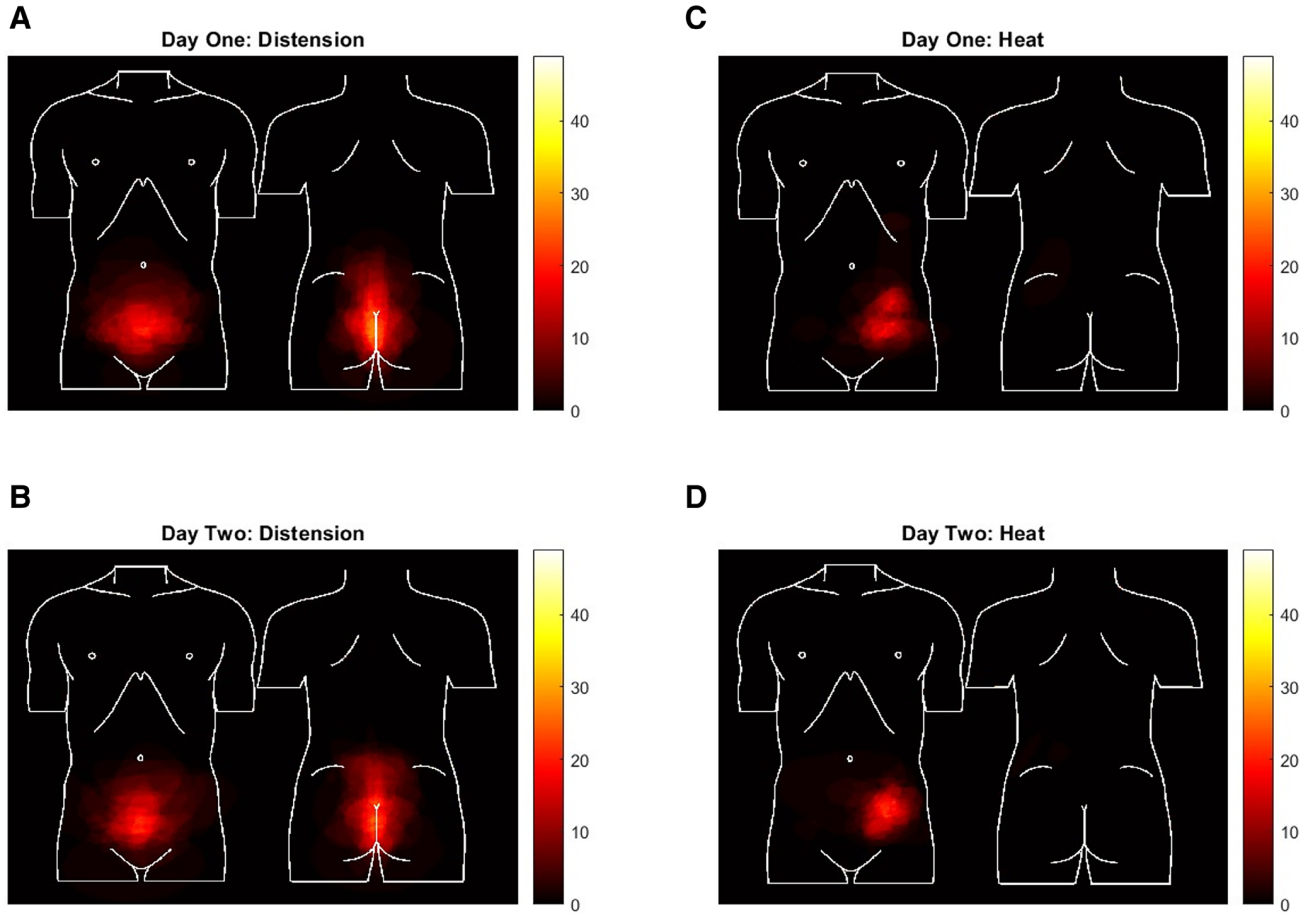
Visualisation of body maps capturing pain areas for evoked visceral (A, B) and somatic pain (C, D) assessed on study days 1 and 2. Results are visualised as an overlay of all digitised sketches; the colour scale represents the number of participants that indicated the presence of pain in the respective body area (black = *N* (0), white = *N* (49)).

Pain drawings were digitised with Inkscape (version 1.1.2) at 3509 × 2550 pixels. Following established procedures (Gholamrezaei et al. 2021), shaded regions (RGB: 255, 0, 0) were extracted in MATLAB (R2022a) to obtain pixel counts, which were log‐transformed prior to analysis to approximate normality. Pain locations were categorised for somatic pain as ‘local’ at the anterior abdomen at the thermode site, and for visceral pain as ‘local’ at the posterior anorectal region, as well as ‘referred pain’ at the anterior abdomen, consistent with IBS‐related referral patterns (Mertz et al. 1995), and ‘total area’ (sum of local and referred). Spatial distributions were visualised using overlapping pixel heatmaps and contour plots to illustrate the relative frequency of pain locations across participants (Figure 1).

### 2.5 Pain Ratings

After each noxious stimulation phase on study day 1, participants rated overall pain intensity and unpleasantness for both visceral and somatic stimuli using digitised 0–100 mm VAS (‘Overall, how painful/unpleasant did you find the distension/heat stimuli you just experienced?’, anchored ‘not at all’ to ‘very painful/unpleasant’). For the present analyses, ratings from all stimulation phases were averaged to derive each participant's overall pain intensity and unpleasantness experience for the entire study day. This approach captures the full variability of pain responses and provides a cumulative measure that corresponds to the whole‐day recall reflected in the body maps. The use of such integrated, retrospective ratings aligns with our rationale, as clinical pain assessments typically rely on patients' global retrospective evaluations rather than moment‐to‐moment pain reports that emphasise sensory‐discriminative rather than affective‐motivational components of pain experience more relevant to the burden of chronic pain (Bolton 1999; Jensen et al. 2012).

### 2.6 Questionnaires Assessing Psychological States and Traits

To explore psychological factors relevant to pain experience and pain recall, we used data from the battery of validated questionnaires participants completed prior to the experimental sessions. The German version of the Hospital Anxiety and Depression Scale (HADS) was administered to assess current levels of anxiety and depression (Laux et al. 2013); the Perceived Stress Scale (PSS) measured subjective stress over the past month (Cohen et al. 1983); the Pain Catastrophizing Scale (PCS) assessed maladaptive cognitive patterns, including rumination, magnification and helplessness (Sullivan et al. 1995); the Somatosensory Amplification Scale (SSAS) evaluated interoceptive sensitivity and bodily vigilance (Barsky et al. 1990); the Big Five Inventory‐10 (BFI‐10) quantifies neuroticism, extraversion, openness, agreeableness, and conscientiousness (Rammstedt and John 2007); the Behavioural Inhibition System (BIS) and Behavioural Activation System (BAS) scales measured approach‐ and avoidance‐related motivational tendencies (Carver and White 1994); emotional states and affective symptom traits were additionally captured using the State–Trait Anxiety–Depression Inventory (STADI, Laux et al. 2013). Collectively, these measures provided a comprehensive assessment of cognitive, affective, and personality factors that may contribute to interindividual variability or to modality‐specific differences in the spatial characteristics of recalled experimental pain.

### 2.7 Statistical Analyses

Unless otherwise specified, descriptive data are presented as mean ± standard error of the mean (SEM).

Differences in pain areas between visceral and somatic pain were assessed using two complementary statistical models. Firstly, total log‐transformed pain area was analysed using a two‐way repeated‐measures analysis of variance (rmANOVA), with pain modality (visceral, somatic) and time (day 1, day 2) as within‐subject factors.

Secondly, regional log‐transformed pixel counts were analysed using a linear mixed‐effects model to compare anterior and posterior pain distribution while accommodating missing anterior pain‐area data. Modality (visceral, somatic), time (day 1, day 2), and location (anterior, posterior) were included as fixed effects, with participant‐specific random intercepts accounting for within‐subject dependence. Fixed effects were tested using Type III Wald F‐tests with Satterthwaite‐approximated degrees of freedom, as implemented in the lmerTest package (Kuznetsova et al. 2017). Partial η^2^ was calculated using eta_squared from the effectsize package (Ben‐Shachar et al. 2020). Post hoc contrasts were computed using the emmeans package (Lenth and Piaskowski 2026) with Holm adjustment (Holm 1979).

The reproducibility of recalled pain areas across one week (day 1, day 2) was evaluated using intraclass correlation coefficients (ICC) calculated with a two‐way random‐effects model for absolute agreement. Both single‐measure and average‐measure ICCs were reported to reflect the reliability of individual and aggregated ratings, respectively. ICC values were interpreted according to Koo and Li (2016): < 0.50 = poor, 0.50–0.75 = moderate, 0.75–0.90 = good, and > 0.90 = excellent reliability. Pearson correlation coefficients were computed to examine (1) relationships between visceral and somatic pain areas, (2) associations of pain area with VAS ratings of intensity and unpleasantness at day 1, and (3) relationships with psychological state and trait variables. For referred pain area, we descriptively report frequencies, complemented by student's *t*‐tests comparing participants with and without referred pain with respect to pain unpleasantness, pain intensity, and trait and state anxiety. For *t*‐tests, effect sizes (Cohen's d) and 95% confidence intervals of the mean differences were reported.

Welch's correction was applied when the assumption of homogeneity of variance was violated. Bayesian linear regression was conducted in JASP (version 0.19.3) to identify potential predictors of visceral pain area, allowing for direct quantification of parameter uncertainty via probability distributions. Variables with significant correlations after Bonferroni correction for multiple comparisons (*α* = 0.003) were included in the regression. Default Cauchy priors (scale = 0.354) were used to regularise estimates and reduce the risk of overfitting. Model fit and predictive value were evaluated using Bayes factors (BF₁₀), posterior means with 95% credible intervals (CrI), and Bayesian R^2^.

## 3 Results

### 3.1 Pain Areas Based on Analysis of Body Maps in Response to Evoked Visceral and Somatic Pain

As shown in Figure 1, all participants indicated visceral pain arising from rectal distension on body maps on day 1 (49/49). Local pain in the posterior anorectal region was marked by 45 of 49 participants (91.8%), and referred pain in the anterior abdominal region by 33 participants (67.3%) (Figure 1A). On day 2, a similar pattern was observed, with 45 of 49 participants again indicating recalled local pain and 31 of 49 indicating referred pain (Figure 1B). For somatic pain induced by thermal cutaneous stimuli, all participants marked local pain at the thermode site on the left anterior abdomen in a very similar area on day 1, and one person additionally indicated pain on the back (Figure 1C). On day 2, 47 participants (95.9%) recalled pain at the same site, two reported no pain, and two additionally indicated pain on the back (Figure 1D).

**FIGURE 2:**
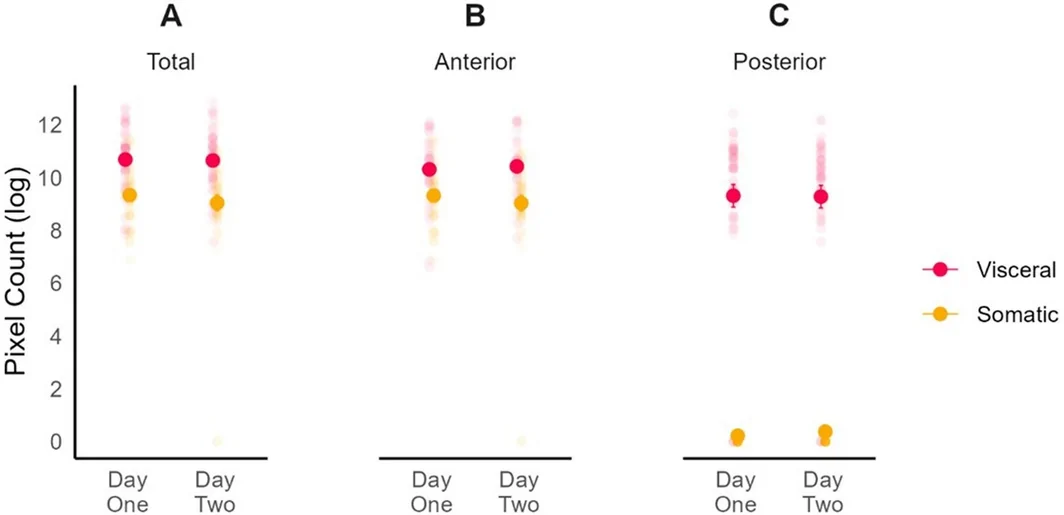
Spatial extent of visceral and somatic pain immediately after stimulation and at 1‐week recall. (A) Log‐transformed total pixel counts; (B, C) log‐transformed anterior and posterior pixel counts. Total area in panel A was derived from the complete drawing and is not the sum of panels B and C. Large dots indicate means, small pale dots individual observations, and error bars standard errors. Zero counts were omitted from the log‐scale display; displayed means and standard errors therefore describe non‐zero observations only. For posterior somatic pain, the small numbers of non‐zero observations precluded meaningful summary estimates. Inferential results reported in the text are based on the linear mixed‐effects model. Non‐zero observations at immediate/recall assessment were: Total visceral, 49/49; total somatic, 49/47; anterior visceral, 33/31; anterior somatic, 49/47; posterior visceral, 45/45; and posterior somatic, 1/2.

The rmANOVA of log‐transformed total pixel counts with modality and time as within‐subject factors revealed a main effect of pain modality, with larger pain areas for visceral compared to somatic pain (Figure 2A; F (1, 46) = 88.61, *p* < 0.001, η_p_^2^ = 0.658). Bonferroni‐adjusted follow‐up comparisons confirmed this difference on day one (MD = 1.39, 95% CI [1.066, 1.718], *p* < 0.001, d = 1.26) and day two (MD = 1.22, 95% CI [0.941, 1.499], *p* < 0.001, *d* = 1.28; Figure 2A). Pain areas were stable across study days for both modalities, as indicated by the absence of a main effect of time (F (1, 46) = 0.23, *p* = 0.63, η_p_^2^ = 0.005) and the absence of a modality × time interaction (F (1, 46) = 2.12, *p* = 0.15, η_p_^2^ = 0.045).

To examine spatial pain distribution, we analysed anterior and posterior log‐transformed pain areas using a linear mixed‐effects model. Significant main effects were found for modality (Figure 2B,C; F (1, 312) = 629.64, *p* < 0.001, ηp^2^ = 0.669) and location (F (1, 312) = 600.01, *p* < 0.001, ηp^2^ = 0.658), together with a significant modality × location interaction (F (1, 312) = 368.15, *p* < 0.001, ηp^2^ = 0.542). No effects involving time were observed (all *p* > 0.469). Post hoc comparisons showed larger visceral than somatic pain areas in both anterior (MD = 1.20, 95% CI [0.41, 1.99], *p* < 0.001) and posterior regions (MD = 9.00, 95% CI [8.31, 9.69], *p* < 0.001). Within modalities, the anterior region showed larger pain areas than the posterior region for both visceral (MD = 1.08, 95% CI [0.29, 1.87], *p* = 0.001) and somatic pain (MD = 8.88, 95% CI [8.19, 9.57], *p* < 0.001).

### 3.2 Correlational Analyses Within and Across Pain Modalities

**FIGURE 3:**
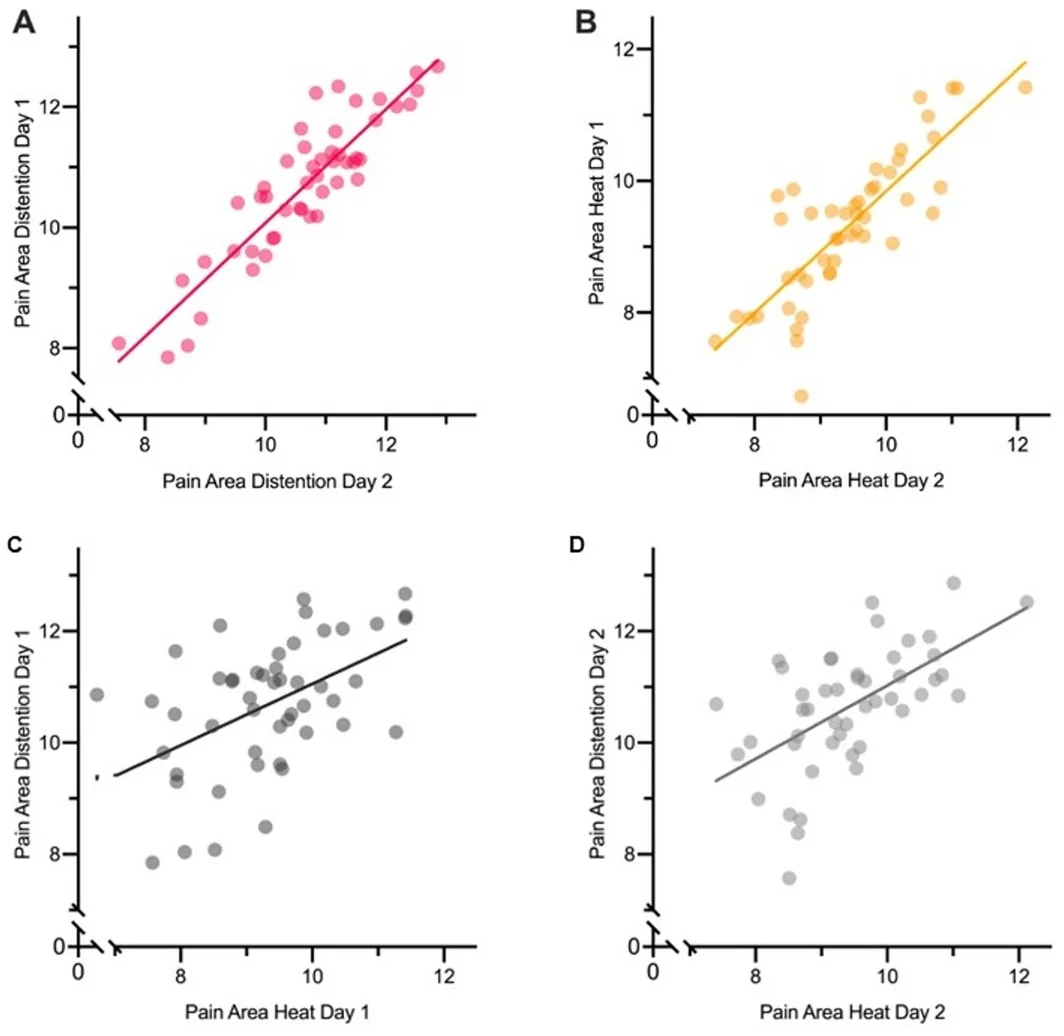
Scatter plots visualising correlations of total pain area across study days within each pain modality for visceral pain (A) and somatic pain (B), and between pain modalities on day 1 (C) and day (2). All correlational analyses revealed *p* < 0.05.

Correlational analyses within each pain modality across the two study days revealed strong positive associations between the total pain area drawn immediately after noxious stimulation on day 1 and the delayed recall pain area drawn at the beginning of day 2 (visceral: *r* = 0.902, *p* < 0.001; Figure 3A; somatic: *r* = 0.825, *p* < 0.001; Figure 3B). Intraclass correlation coefficients (ICC) corroborated these findings, demonstrating excellent agreement across timepoints. For visceral pain, the ICC for single measures was 0.903 (95% CI [0.834, 0.944]) and 0.949 (95% CI [0.909, 0.971]) for average measures. For somatic pain, the ICC for single measures was 0.818 (95% CI [0.696, 0.894]) and 0.900 (95% CI [0.821, 0.944]) for average measures. All ICCs were statistically significant (*p* < 0.001). Correlational analyses across modalities showed moderate positive associations between total visceral and somatic pain areas on both days (day 1: *r* = 0.504, *p* < 0.001; Figure 3C; day 2: *r* = 0.584, *p* < 0.001; Figure 3D).

### 3.3 Body Maps and VAS Pain Measures

Analysis of averaged VAS ratings obtained on day 1 showed that visceral pain was perceived as more intense than somatic pain (visceral: 58.8 ± 2.3; somatic: 46.3 ± 3.2; MD = 12.56, 95% CI [5.03, 20.09]; t (48) = 3.35, *p* = 0.002, d = 0.48). Visceral pain was also rated as more unpleasant (visceral: 67.8 ± 3.0; somatic: 41.6 ± 3.2; MD = 26.13, 95% CI [17.85, 34.41]; t (48) = 6.35, *p* < 0.001, *d* = 0.91). Correlational analyses summarised in Table 1 indicated that VAS ratings were generally unrelated to pain areas derived from body maps on day 1; neither unpleasantness nor intensity ratings showed meaningful associations with local or total pain areas for either modality. The only exception was a moderate positive correlation between visceral pain intensity and referred pain area on day 1, suggesting that higher visceral pain intensity was associated with larger referred pain distributions.

**TABLE 1:** Results of correlational analyses between pain area and VAS pain ratings.

Modality	VAS outcome	Pain area	R	P
Visceral	Intensity	Total	0.255	0.077
		Local	0.096	0.532
		Referred	**0.431**	**0.012**
	Unpleasantness	Total	−0.035	0.809
		Local	−0.152	0.319
		Referred	0.153	0.395
Somatic	Intensity	Total/local	0.085	0.561
	Unpleasantness	Total/local	0.105	0.472

*Note:* Bold values indicate statistically significant correlations (p < 0.05).

Additionally, neither somatic nor visceral pain area was significantly associated with the corresponding pain threshold or individually calibrated VAS50 stimulation level (all *p* ≥ 0.214).

### 3.4 Correlations and Bayesian Regression With Psychological States and Traits

To explore the contribution of psychological state and trait measures, we examined correlations between total pain area for visceral and somatic pain at day 1 and questionnaire scores (Table 2). For visceral pain, after Bonferroni correction for multiple correlations (*p* < 0.003), larger pain areas were associated with higher neuroticism, whereas smaller visceral pain areas correlated with higher levels of extraversion, agreeableness, and behavioural inhibition. Associations for somatic pain were less frequent; the most prominent correlations were negative, indicating that higher behavioural inhibition and reward responsiveness were linked to smaller, and neuroticism was associated with higher somatic pain areas.

**TABLE 2:** Correlational analyses between total pain area and psychological measures.

Variable	Mean ± SEM	Visceral pain area		Somatic pain area	
		*R*	*p*	*R*	*p*
State anxiety (STADI‐S)	14.88 ± 0.55	−0.224	0.126	0.019	0.899
State depression (STADI‐S)	17.09 ± 0.40	−0.202	0.179	−0.168	0.265
Trait anxiety (STADI‐T)	17.33 ± 0.67	0.357	0.020	0.279	0.074
Trait depression (STADI‐T)	15.74 ± 0.61	0.257	0.100	0.067	0.674
Perceived stress (PSS)	14.54 ± 0.82	0.207	0.188	0.186	0.238
Pain catastrophizing (PCS)	17.70 ± 1.49	0.268	0.094	0.129	0.428
Somatosensory amplification (SSAS)	27.18 ± 0.81	0.260	0.088	0.156	0.311
Neuroticism (BFI‐10)	5.63 ± 0.30	**0.449**	**0.003**	**0.449**	**0.003**
Extraversion (BFI‐10)	7.22 ± 0.32	**−0.620**	**< 0.001**	−0.275	0.082
Openness (BFI‐10)	7.29 ± 0.29	0.440	0.004	0.174	0.277
Agreeableness (BFI‐10)	7.17 ± 0.22	**−0.468**	**0.002**	−0.223	0.161
Conscientiousness (BFI‐10)	7.20 ± 0.25	−0.061	0.706	−0.251	0.113
BIS	14.78 ± 0.55	**−0.605**	**< 0.001**	**−0.496**	**< 0.001**
BAS Drive	7.68 ± 0.28	0.172	0.281	0.145	0.366
BAS Fun Seeking	7.56 ± 0.29	0.156	0.331	−0.139	0.386
BAS Reward Responsiveness	8.93 ± 0.35	−0.083	0.607	−0.337	0.031

*Note:* Bold values indicate statistically significant correlations (p ≥ 0.003).

Abbreviations: BAS, Behavioural Activation System; BFI‐10, Big Five Inventory‐10; BIS, Behavioural Inhibition System; p, exact *p*‐values of correlational analyses; PCS, Pain Catastrophizing Scale; PSS, Perceived Stress Scale; r, Pearson's r; SSAS, Somatosensory Amplification Scale; STADI, State–Trait Anxiety Depression Inventory (S, state version; T, trait version).

To further identify predictors of total visceral pain area, we conducted a Bayesian linear regression including all variables that showed significant correlations. The best‐fitting model included the personality traits extraversion (BFI‐10) and behavioural inhibition tendency (BIS). This model accounted for 49.8% of the variance in total visceral pain area (*R* ^2^ = 0.498) and showed moderate evidence in favour of the alternative over the null model (BF₁₀ = 10.34). Among the predictors, extraversion showed the strongest evidence for inclusion (BF_incl_ = 13.96, *β* = −0.20, 95% CrI [−0.35, 0.00]), followed by behavioural inhibition tendency (BF_incl_ = 5.80, *β* = −0.10, 95% CrI [−0.22, 0.00]). Although their credible intervals bordered on zero, their inclusion Bayes factors provided moderate to strong evidence for their contribution to the model. Neuroticism (BF_incl_ = 0.71, 95% CrI [−0.21, 0.14]) and agreeableness (BF_incl_ = 1.06, 95% CrI [−0.30, 0.08]) yielded BF_incl_ near 1, and both credible intervals included zero, providing only weak or anecdotal evidence for their predictive value. Equivalent Bayesian analysis was not conducted for somatic pain area, as too few correlations with psychological variables emerged in the present data.

### 3.5 Referred Pain Subgroup Analysis

With over two‐thirds of participants reporting referred pain on day 1, we compared participants with and without referred pain regarding pain intensity, pain unpleasantness, trait anxiety, and state anxiety. Participants with referred visceral pain (defined as a non‐zero pain area in the anterior referred‐pain region) reported higher pain unpleasantness than those without (VAS: mean = 72.1, SD = 20.13 mm vs. mean = 58.73, SD = 20.69 mm; MD = 13.39, 95% CI [0.94, 25.84], t(47) = 2.16, *p* = 0.036, *d* = 0.66), whereas no significant difference was observed for pain intensity (VAS: mean = 60.89, SD = 14.85 mm vs. mean = 54.62, SD = 18.94 mm; MD = 6.27, 95% CI [−3.70, 16.23], t(47) = 1.26, *p* = 0.212, *d* = 0.39). Regarding anxiety, individuals with referred visceral pain exhibited higher trait anxiety (STADI‐T: mean = 18.28, SD = 4.67 vs. mean = 15.23, SD = 2.65; MD = 3.05, 95% CI [0.74, 5.35], t(37.49) = 2.68, *p* = 0.011, *d* = 0.73), while no difference was found for state anxiety (STADI‐S for those reporting referred pain: mean = 14.85, SD = 3.85 vs. for those who did not: mean = 14.93, SD = 3.81; MD = −0.08, 95% CI [−2.49, 2.32], t(46) = −0.071, *p* = 0.944, *d* = −0.02).

## 4 Discussion

Visceral pain is highly prevalent and clinically burdensome, particularly in gut‐brain axis disorders such as irritable bowel syndrome or inflammatory bowel disease. Its diffuse topography, complex neurophysiology, and integration with affective and cognitive processes hinder assessment and management effectively. Previous work has demonstrated the specificity of visceral versus somatic pain perception using pain ratings and neural activation patterns (Grundy et al. 2019). The poorly localisable nature of visceral pain is a hallmark of its interoceptive character, yet its spatial representation remains understudied. Building on body‐map comparisons of oesophageal pain and cutaneous heat pain applied to the overlying chest area (Strigo et al. 2002), we combined lower gastrointestinal visceral and lower abdominal cutaneous heat pain in healthy volunteers to investigate pain areas, including their spatial distribution, stability over one week, and associations with pain ratings and psychological traits. Our results characterise the spatial distinctiveness of visceral pain relative to somatic pain and provide an understanding of modality‐specific nociception for research and clinical assessment.

### 4.1 Pain Areas

Findings confirmed that acute visceral pain was more unpleasant than intensity‐matched somatic pain, independent of stimulus duration (Chae et al. 2022; Koenen et al. 2017; Strigo et al. 2002; Van Oudenhove et al. 2020), and demonstrated reproducible differences in recalled spatial characteristics. Visceral pain areas were larger and more diffuse than somatic pain, consistent with sparse polysynaptic visceral innervation and spinal viscerosomatic convergence (Farmer et al. 2014; Luz et al. 2015; Sikandar and Dickenson 2012). Body maps also revealed stable inter‐individual differences in pain distribution. Visceral and somatic pain areas showed high reproducibility when recalled after one week, in line with prior work on musculoskeletal and cutaneous pain (Barbero et al. 2015; Galve Villa et al. 2022; Leoni et al. 2017), supporting spatial pain memory accuracy and indicating that body maps capture enduring spatial representations rather than transient perceptual artefacts (Margolis et al. 1988), even when based on recall. The temporal stability indicates that individual differences in pain area may reflect trait‐like variations in central processing and perceptual organisation across modalities. Finally, modality‐specific differences in spatial pain distribution align with prior work suggesting that visceral and somatic pain are related yet distinct modalities. Moderate cross‐modal correlations in pain thresholds (Horing et al. 2013), differences in perceptual and affective responses (Aulenkamp et al. 2026; Koenen et al. 2017; Strigo et al. 2002), distinct but overlapping neural processing (Dunckley, Wise, Fairhurst, et al. 2005), and differential modulation by neuroendocrine stress responses, including cortisol (Benson et al. 2019), collectively support shared and modality‐specific mechanisms.

### 4.2 The Link Between Pain Intensity and Area

Pain areas did not correlate with intensity or unpleasantness ratings, supporting spatial pain representation as a distinct perceptual dimension largely independent of pain magnitude (Adamczyk et al. 2025; Galve Villa et al. 2022). This dimension may reflect stable cortical representation fields or trait‐like mapping tendencies rather than stimulus strength per se. However, calibration to moderate pain (~50 mm VAS) restricted variance in pain ratings and may have limited the detection of associations; pain area may differ under experimentally varied stimulus intensities. Also, while this dissociation could be partly influenced by methodological factors, given that VAS ratings were averaged across the entire study day whereas body maps were completed at the day's conclusion, reducing temporal correspondence, it was observed for both visceral and somatic modalities. If so, this suggests that the spatial dimension of pain recall is a distinct facet of both visceral as well as somatic pain, capturing clinically relevant aspects of pain reporting that standard ratings may overlook.

Although somatic cutaneous pain was localised, the mapped areas frequently extended beyond the thermode contact area. As the markings remained contiguous with the stimulation site, this extension differed from referred pain and resembled patterns described as pain radiation in previous experimental studies (Adamczyk et al. 2025; Price et al. 1978; Quevedo and Coghill 2009).

Interestingly, visceral referred pain was the only body map–derived measure associated with pain intensity, highlighting its potential as a marker of central amplification rather than a simple reflection of peripheral spread (Arroyo‐Fernández et al. 2020; Graven‐Nielsen 2006; Woolf 2011). Referred visceral pain occurred in approximately two‐thirds of participants, most commonly in the anterior abdomen, corroborating previous experimental findings using rectal or oesophageal distension (Lee et al. 2012; Pedersen et al. 2004; Strigo et al. 2002) or even somatic stimulation such as hypertonic saline injection (Laursen et al. 1997). Mechanistically, referred pain is thought to arise from viscerosomatic convergence and altered somatosensory integration, potentially involving unmasking of previously silent synaptic connections or enhanced responsiveness of second‐order dorsal horn neurons (Arendt‐Nielsen and Svensson 2001; Graven‐Nielsen 2006; Laursen et al. 1997; Pedersen et al. 2004). Participants reporting referred pain also exhibited higher unpleasantness ratings and elevated trait anxiety. Together, these findings suggest that referred visceral pain serves not only as a marker of nociceptive intensity but also as a potential indicator of central amplification mechanisms, involving emotional reactivity and attentional modulation. Comparable central mechanisms have also been described in models of musculoskeletal referred pain (Graven‐Nielsen and Arendt‐Nielsen 2010), while musculoskeletal pain often shows broader and referred distributions on body maps, particularly under chronic conditions (Barbero et al. 2015; Egloff et al. 2012; Visser et al. 2016). Understanding these mechanisms may be particularly relevant in clinical populations, where referred pain is often more pronounced (Woolf 2011) and may indicate heightened central sensitization or an increased risk of chronicity, especially in individuals with multiple comorbidities.

### 4.3 Psychological Traits and States and Its Link to Pain Distribution

Several traits and states have been linked to symptom generation and treatment outcomes in psychobiological models of brain–gut interactions (Guadagnoli et al. 2025) as well as in general models of pain chronicity (Vlaeyen et al. 2016). To provide a foundation for such analyses in patient populations, we examined correlations between pain areas and relevant psychological traits and states in our healthy volunteer sample to elucidate potential mechanisms underlying inter‐individual differences in pain recall within each pain modality. Interestingly, visceral pain areas were more strongly associated with psychological traits than somatic areas, in line with neurophysiological evidence that visceral afferents preferentially project to limbic and paralimbic structures involved in emotion and interoception (Benson et al. 2019; Moloney et al. 2016; Paine, Kishor, et al. 2009; Paine, Worthen, et al. 2009).

In bivariate analyses, larger visceral pain areas correlated with higher neuroticism, whereas smaller areas were associated with higher extraversion, agreeableness, and behavioural inhibition. In multivariate Bayesian regression models, extraversion and behavioural inhibition were the strongest predictors. Extraversion has been linked to greater pain tolerance and reduced pain intensity in both experimental and clinical settings (Harkins et al. 1989; Ramírez‐Maestre et al. 2004), and heightened behavioural inhibition was reportedly associated with greater pain interference and avoidance behaviour in chronic pain patients (Jensen et al. 2017; Turner et al. 2021). These findings extend evidence that visceral pain is particularly susceptible to modulation by fear and expectancy‐related mechanisms, including nocebo effects (Aulenkamp et al. 2026; Koenen et al. 2017).

Neuroticism correlated positively with visceral pain area in bivariate analyses but lost significance in multivariate models. Individuals high in neuroticism may exhibit heightened emotional reactivity and hypervigilance to internal bodily signals, which could amplify pain perception and expand its cortical representation (Goubert et al. 2004).

## 5 Limitations

The multimodal, within‐subject design allowed direct comparisons across pain modalities and repeated assessments, enhancing internal validity. The excellent one‐week reliability of body maps supports their utility for longitudinal studies. Limitations include the use of a two‐dimensional torso representation, which may not fully capture diffuse visceral sensations; the comparison of absolute spatial pain maps without accounting for interindividual differences in the precise anatomical location of pain; exploratory analyses with moderate sample size (*N* = 49), limiting precision in multivariate models; and the exclusive inclusion of healthy young adults, restricting generalisability to clinical populations or older individuals with modality‐specific pain processing changes (El Tumi et al. 2017). Moreover, questionnaire scores were below established clinical thresholds, calling for replication in cohorts with clinically relevant scores to consolidate this proposition.

## 6 Conclusions

Our findings demonstrate that body maps reliably capture spatial characteristics of pain, distinguish interoceptive from exteroceptive modalities, and capture modality‐specific associations with psychological traits. Visceral pain showed broader spatial distribution, greater unpleasantness, and stronger associations with personality traits, particularly behavioural inhibition and extraversion. Interoceptive pain from the lower abdomen differs from somatic pain not only in intensity and unpleasantness but also in spatial distribution and psychological modulation—insights not captured by ratings alone. In the context of visceral pain assessment, use of anterior and posterior body maps could improve detection of referred areas, allowing precise monitoring of centralization trends and informing mechanism‐based interventions, including cognitive‐behavioural and pharmacological treatment approaches. Combined with psychological assessment, body maps may help stratify patients with gut–brain interaction disorders into subgroups dominated by central amplification versus peripheral input. The independence of spatial and intensity dimensions underscores the value of multimodal assessment in research and clinical practice. These findings pave the way for future studies on pain modulation, treatment sensitivity, and longitudinal monitoring using spatial measures, providing a translational bridge from experimental findings to clinical assessment and management of visceral pain.

## Author Contributions

This study was designed by Sigrid Elsenbruch. The experiments were performed by Catrin Guddat, Robert J. Pawlik and Jana L. Aulenkamp. The data were analysed by Catrin Guddat and Jana L. Aulenkamp, and the results were critically examined by all authors. Catrin Guddat had a primary role in preparing the manuscript, which was edited by Jana L. Aulenkamp, Sigrid Elsenbruch and Thomas Graven‐Nielsen. All authors have approved the final version of the manuscript and agree to be accountable for all aspects of the work.

## Funding

This work was funded by the Deutsche Forschungsgemeinschaft (DFG, German Research Foundation) – Project‐ID 422744262—TRR 289 (Deutsche Forschungsgemeinschaft, DFG). Author JLA was funded by the Clinician Scientist Program UMEA of the University Hospital Essen (FU 356/12–2). TGN is affiliated with the Center for Neuroplasticity and Pain (CNAP) supported by the Danish National Research Foundation (DNRF121). TGN receives funding from the Lundbeck Foundation (R441‐2023‐232).

## Conflicts of Interest

The authors declare no conflicts of interest.

## Data Availability

Data will be made available from the corresponding author upon request Anonymized data: https://osf.io/ng98j.
